# COPD and Cardiovascular Diseases: Biomarker-Guided Stratification and Therapeutic Perspectives

**DOI:** 10.3390/jcm15010049

**Published:** 2025-12-21

**Authors:** Melika Valizadeh, Jens-Ulrik Jensen, Alexandr Ceasovschih, Alexandru Corlateanu, Pradeesh Sivapalan

**Affiliations:** 1 Pulmonary Rehabilitation Research Center (PRRC), National Research Institute of Tuberculosis and Lung Diseases (NRITLD), Shahid Beheshti University of Medical Sciences, Tehran 19839-63113, Iran; 2 Department of Clinical Medicine, Faculty of Health and Medical Sciences, University of Copenhagen, DK-2200 Copenhagen, Denmark; 3 Copenhagen Respiratory Research, Department of Medicine, Copenhagen University Hospital—Herlev and Gentofte, DK-2630 Copenhagen, Denmark; 4 Faculty of Medicine, Grigore T. Popa University of Medicine and Pharmacy, 700115 Iasi, Romania; 5 Head of Department of Respiratory Medicine and Allergology, Nicolae Testemitanu State University of Medicine and Pharmacy, MD-2004 Chisinau, Moldova; alexandru.corlateanu@usmf.md

**Keywords:** chronic obstructive pulmonary disease, COPD, cardiovascular diseases, biomarkers, inflammation

## Abstract

Chronic obstructive pulmonary disease (COPD) is a progressive and preventable respiratory disorder associated with a significantly increased risk of cardiovascular disease (CVD), including ischemic heart disease, heart failure, pulmonary hypertension, and arrhythmias. The association is bidirectional, with CVD adversely affecting COPD prognosis and vice versa. Beyond shared risk factors such as smoking, aging, and socioeconomic status, COPD itself, through mechanisms including systemic inflammation, oxidative stress, hypoxia, and hyperinflation, contributes independently to cardiovascular morbidity and mortality. Recent research has focused on identifying circulating biomarkers that can aid in early detection, risk stratification, and treatment optimization for patients at risk of both conditions. This review summarizes current evidence on the pathophysiological mechanisms linking COPD and CVD, highlights emerging biomarkers with potential prognostic utility for major adverse cardiovascular events (MACE) and all-cause mortality, and it discusses clinical and research implications for biomarker-guided, personalized treatment strategies.

## 1. Introduction

Chronic obstructive pulmonary disease (COPD) is a progressive chronic lung disease characterized by airflow limitation and it leads to a higher rate of comorbidity and mortality [1]. It has been shown that COPD patients are two to five times more likely to suffer from cardiovascular diseases, like ischemic heart disease (IHD), cardiac dysrhythmias, heart failure (HF), and pulmonary hypertension, in addition to peripheral vascular diseases [2]. This increased risk is seen even after adjusting for risk factors such as sex, age, and dyslipidemia [3]. In fact, mortality due to cardiovascular diseases (CVD) often exceeds mortality due to respiratory failure in these patients [4]. The reverse association is also observed. For example, the prevalence of COPD among patients with HF is estimated at 13–39% [5], and the presence of COPD worsens the prognoses of patients hospitalized for HF [6]. Similarly, COPD prevalence among IHD patients is estimated at approximately 12% [7]. Atrial fibrillation (AF), the most common supraventricular arrhythmia in COPD, is both a risk factor for exacerbation and a complication that can be triggered by the acute exacerbation of COPD [8]. It has been observed that the prevalence of coronary heart disease is higher in males with COPD compared to females, and congestive heart failure is associated with a greater risk of mortality in these individuals [9]. According to the World Health Organization, the prevalence of cardiovascular disease and COPD is higher in the elderly and is associated with a higher rate of death in this group [10]. Although more evidence is still needed, it has been suggested that cardiovascular diseases can occur in different stages of COPD [11].

The mechanisms linking COPD and CVD are complex and multifactorial [12]. Although both diseases share common and well-known risk factors including smoking, age, and socioeconomic level [5], studies have shown that airflow limitation is an independent risk factor for CVD [13,14].

Different pathophysiological mechanisms, including systemic inflammation, oxidative stress, hypoxia, and hyperinflation, can explain the close association between COPD and CVD [15].

This review aims to summarize the current evidence on the pathophysiological mechanisms linking COPD and cardiovascular disease and discuss emerging biomarkers for early detection in patients at risk of both diseases and risk stratification.

## 2. Methodology

This narrative review was conducted to integrate and evaluate the current evidence on mechanisms linking COPD and cardiovascular disease, and it highlights biomarker-guided stratification and therapeutic perspectives for both diseases.

A comprehensive literature search was performed using PubMed, Web of Science, and Scopus for articles published from 2000 to 2025. The key search terms included (“chronic obstructive pulmonary disease” OR “COPD”) AND (“cardiovascular disease” OR “CVD”).

We included observational studies, randomized clinical trials, and seminal review articles. We excluded case reports, editorials, and non-English articles.

## 3. Linking Mechanisms

Systemic inflammation is a well-known feature of COPD. Recurrent exposure to inhaled pollutants, such as cigarette smoke and infections, can contribute to an inflammatory process in the lungs [16]. These processes are associated with greater morbidity and mortality in patients with COPD [17]. The possibility has been raised that local inflammation in the airways can lead to the overspill of inflammatory mediators such as serum cytokines and chemokines into systemic circulation [1]. In COPD, persistent inflammation involves elevated proinflammatory cytokine levels and oxidative stress [18]. Multiple immune cell types, neutrophils, macrophages, and lymphocytes are engaged [19], with contributions from innate and adaptive immunity [4]. This immune activation alters the Th1/th2 balance and affects macrophages and dendritic cell function [18].

In COPD patients, chronic inflammation not only affects the lung parenchyma and airways but also different systems, including the vascular endothelium [5]. These mechanisms increase leukocyte migration from the endothelium and can facilitate the upregulation of intercellular adhesion molecules (ICAM), and accordingly, increased levels of ICAM1 are associated with decreased pulmonary function and emphysema severity [20,21]. A CVD prognosis has been shown to be associated with increased levels of inflammatory markers such as ICAM1, interleukin 6(IL-6), and C-reactive protein (CRP) [22].

Low-grade inflammation determines CVD risk by increasing the rate of formation and rupture of atherosclerotic plaques, the proliferation of smooth muscle cells and, consequently, arterial stiffness, promoting platelet aggregation, endothelial dysfunction, and, finally, a reduction in endothelial progenitor cells (EPCs) [15].

Endothelial dysfunction has been demonstrated to be a key factor in the atherogenesis process in COPD and also an important factor in CVD pathogenesis [23,24]. A decrease in nitric oxide (NO) concentrations is a hallmark of endothelial dysfunction and can stem from either reduced activity of endothelial NO synthase (eNOS) or diminished NO bioavailability [25].

It appears that oxidative stress and inflammation result in a reduction in NO bioavailability and endothelial dysfunction, and this pathway likely plays a fundamental role in ED in COPD patients [26,27]. There is evidence that activation of the renin-angiotensin system also plays a role in the endothelial dysfunction seen in COPD patients [25]. Endothelial dysfunction can be worsened by cigarette smoke through mechanisms such as increased alveolar-capillary permeability [28].

Since endothelial dysfunction is important in the progression of COPD and the risk of cardiac disease, noninvasive assessment methods such as flow-mediated dilation (FMD) and peripheral artery tonometry (PAT) are useful in these patients [23].

Arterial stiffness is likely caused by an imbalance in matrix metalloproteases and is associated with elastin degradation, which leads to elastin destruction in the vasculature and pulmonary emphysema [29], and it has been suggested that it plays a role as a predictor of cardiovascular events [30]. Since data have shown that arterial stiffness is independently associated with COPD and, consequently, a higher risk of cardiovascular events, arterial pulse-wave velocity (PWV) appears to be a useful method for evaluating arterial stiffness in these patients [2].

Platelet activation may be a link between COPD, inflammation, and cardiovascular disease [31]. The interaction between platelets and inflammatory cells leads to the release of chemokine and the progression of the accumulation of immune mediators, resulting in the formation of atherosclerotic plaques [32]. An observational study showed that platelet activation in patients with more severe COPD and acute exacerbation is associated with a higher risk of cardiovascular comorbidity [33]. Studies have also shown that platelet activation results in structural changes in pulmonary vasculature, which could play a role in the pathogenesis of pulmonary arterial hypertension (PAH) [32,34].

Chronic inflammation also impairs the function, survival, and motility of EPCs, which play a role in vascular endothelium repair [35].

Oxidative stress occurs when there is an imbalance between the production of reactive oxygen species (ROS) and the ability of antioxidants to neutralize their effects [36]. Oxidative stress plays a role in various pathologies, such as arterial hypertension, atherosclerosis, Alzheimer’s disease, diabetes, COPD, and asthma [5]. Following the inhalation of particles or inflammatory mediators, the accumulation of phagocytes in the lower respiratory tract leads to the production of large amounts of ROS. Increased ROS in oxidative stress is a key driver in the progression of COPD and endothelial dysfunction. ROS cause lipid peroxidation via activation of the receptor for advanced glycation end-products (RAGE) and ferroptosis [37,38]. In addition, oxidative stress induces asymmetric dimethylarginine (ADMA) accumulation, which can impair NO bioavailability and increase arginase activity, and this can contribute to endothelial dysfunction and cardiovascular issues in COPD patients [38].

Hypoxia is commonly seen in severe COPD due to a ventilation–perfusion (V/Q) mismatch [39]. Hypoxia can induce systemic inflammation and oxidative stress and trigger the formation of foam cells which accumulate lipids and contribute to development of atherosclerotic plaques and coronary artery disease in these patients [40]. Hypoxia-inducible factors (HIFs) are a family of transcription factors that are actually oxygen sensors and act as regulators of cellular adaptation in response to low oxygen levels [41]. These factors play a role in the development and progression of cardiovascular disease. They can induce angiogenesis and vascular remodeling, such as the pulmonary arterial remodeling in response to chronic hypoxia that leads to PAH [5,42]. It has been suggested that hypoxia-inducible factor 1-alpha upregulation may increase AF risk [43].

Hyperinflation occurs when air becomes trapped in the lungs following exhalation in conditions like COPD [44], and it leads to increased intrathoracic pressure and end expiratory lung volume [2]. Increased intrathoracic pressure inhibits venous return to the right ventricle by compressing the major vessels, and this elevated pressure during expiration causes a decrease in the right ventricle (RV) preload and an increase in pulmonary vascular resistance (PVR) and RV afterload. This condition can lead to RV strain and right side heart dysfunction, and it can further cause left ventricular (LV) dysfunction due to the pressure of the interventricular septum on LV [45,46]. Lukacsovits et al. showed that exercise-induced dynamic hyperinflation in COPD patients worsens cardiovascular adjustment during exercise, which is influenced by stroke volume and cardiac output [47].

Given the prevalence of cardiovascular comorbidity in COPD and the importance of reduced lung function in the development of cardiovascular disease, identifying biomarkers associated with CVD in COPD patients can provide a better understanding of the mechanisms and pathogenesis linking these two diseases, and it can also be used as an early diagnostic tool to identify COPD patients with higher risk of CVD and improve management [1].

## 4. Biomarkers

C-reactive protein (CRP): CRP accelerates the process of eliminating necrotic and apoptotic cells and pathogens by activating the classical complement pathway, and it responds rapidly to inflammatory stimuli [48]. CRP is a potential biomarker of chronic inflammation and atherosclerosis in COPD. It has been observed that decreases in the Tiffeneau–Pinelli index and forced expiratory volume in 1 s (FEV1) are associated with an increased level of high sensitivity to CRP (hs CRP), and the risk of ischemic heart disease is higher in patients with moderate-to-severe obstruction and higher CRP levels [4,49]. A cohort study showed that elevated levels of inflammatory markers such as CRP, fibrinogen, and leukocytes were associated with an increased risk of exacerbation in patients with stable COPD [50]. Therefore, CRP can be used as a predictor for the future exacerbation of COPD. Another study has also shown the usefulness of using CRP as a guiding biomarker in antibiotic treatment in the acute exacerbation of COPD [51]. CRP as a marker of systemic inflammation has also been mentioned in monitoring statin use in COPD. It has been shown that the use of statins in COPD patients with high baseline hsCRP (>3) has been associated with a decrease in mortality [52].

Fibrinogen: A soluble glycoprotein involved in the pathway of the coagulation system, fibrinogen is an acute phase reactant protein that participates in immunoinflammatory responses via binding to inflammatory cells, and it promotes inflammation that has been proposed as a marker of activity in COPD [53]. The IMPACT study demonstrated that fibrinogen can be used as a predictive biomarker for COPD exacerbation [54]. A sub-study of the SUMMIT trial showed that fibrinogen and CRP were associated with an increased risk of mortality in COPD patients; however, it could not show an association between elevated fibrinogen or CRP levels and decreased FEV1, hospitalization, and exacerbation rate [55]. Elevated fibrinogen levels are associated with a higher risk of coronary events in COPD patients [56,57]. A high level of fibrinogen can be a predictor in a patient with a higher risk of COPD exacerbation and cardiovascular and hypercoagulative complications.

Brain-type natriuretic peptide (BNP) and N-terminal proBNP (NT-proBNP): As early and sensitive biomarkers for an HF diagnosis, these peptides are secreted by cardiomyocytes in response to ventricular stretch. Increased levels are seen in right ventricular dysfunction, pulmonary hypertension, and cor pulmonale [58,59]. Natriuretic peptide levels are also increased in COPD patients without HF [60], and this elevation is associated with severity of right ventricular dysfunction, disease activity, rate of exacerbation, and mortality risk [4,61]. In practice, these biomarkers can be used to differentiate the symptoms caused by acute heart failure from COPD exacerbation, a BNP level of <100 pg/mL, and an NT pro BNP level of < 300 pg/mL, which makes acute decompensate heart failure very unlikely. In addition, they are useful for evaluating the response to heart failure treatment as well as identifying cor pulmonale patients and COPD patients with higher mortality risk.

High-sensitivity cardiac troponin T (hs-cTnT): A useful marker for the detection of myocardial injury, hs-cTnT can be influenced by inflammation and right-heart overload [62]. Several studies have shown that the level of cardiac troponin is higher in a patient with stable COPD. Increased inflammatory activity has been proposed as a possible mechanism. Pro-inflammatory cytokines like IL-6 can enhance the permeability of cardiomyocyte cell walls and stimulate troponin release [62,63,64]. It has also been demonstrated that elevated troponin levels in patients hospitalized due to COPD exacerbation are associated with a high mortality rate [65]. Detecting elevated troponin levels in patients with COPD exacerbation is crucial for the accurate monitoring of acute coronary events as well as for the identification of patients at higher risk of complications and mortality.

Vascular endothelial growth factor (VEGF): VEGF is a signaling protein which plays a role in vascular development [66]. It is an important predictor of CVD through angiogenesis, increased migration, and the proliferation of endothelial cells, and it can also play a role as a proinflammatory cytokine by promoting vascular permeability and thrombogenesis [4,67]. Higher VEGF serum levels have been seen in patients with COPD exacerbation. Hypoxia and inflammatory state in COPD patients can activate VEGF and cause cardiovascular or thrombosis events [49,68].

Surfactant protein D (SP-D): SP-D is a protein that plays an important role in regulating immunity and inflammation in the lungs [69]. The ECLIPSE cohort study showed that elevated SP-D levels are associated with an increased risk of exacerbation within the following year. This study also found that SP-D levels are connected to 3-year all-cause mortality [70,71]. SP-D has a dual role in the cardiovascular system, and while it can have an anti-inflammatory role in the coronary arteries, it can also have a pro-inflammatory role leading to atherosclerosis [72]. Although this marker is not currently used clinically, it appears that it can be helpful in determining COPD patients with risk of exacerbation and cardiovascular events due to the underlying inflammatory state of both conditions.

Galectin-3 (Gal-3): Gal-3 is a beta-galactoside binding lectin that relates to kidney disease progression and also participates in various pathways such as inflammation, fibrosis, and myocardial remodeling [73]. It has been suggested that increased levels of Gal-3 may indicate myocardial fibrosis and remodeling in COPD patients with CVD [74]. Gal-3 levels have been proposed as a potential biomarker for COPD exacerbation [75]. Based on a study of 120 stable and pre-COPD patients, it was observed that Gal-3 levels were notably correlated with NT-proBNP levels and negatively correlated with maximal pulse rate during 6-minute walk test [76]. In addition, the study suggested that hs-TnT is a more sensitive biomarker for changes in cardiopulmonary function; however, Gal-3 may be valuable for identifying patients at risk of cardiovascular comorbidity and can be used to monitor the progression of myocardial remodeling and fibrosis. Therefore, it was suggested that monitoring Gal-3 in addition to hs-TnT and NT-proBNP may be a more complete assessment for cardiovascular event risk in COPD patients [76].

Growth differentiation factor-15 (GDF-15): GDF-15 is a cytokine that belongs to the transforming growth factor β superfamily [77]. It is a cellular stress marker whose expression is increased in various pathologic conditions like cellular senescence, ischemia, cancer, and CVD [77,78]. Several studies have suggested that GDF-15 is associated with poor muscle function, and serum GDF-15 levels can be used to assess sarcopenia in COPD patients [78]. Also, comparing COPD patients with healthy and asthmatic individuals showed higher serum GDF15 levels and demonstrated its relationship with lower exercise capacity [79]. In a study of 413 patients with COPD, it was observed that higher levels of GDF-15 were independently associated with increased exacerbation frequency, higher mortality, greater declines in FEV1, and forced vital capacity [80]. In a cohort study that included smokers without CVD, it was found that GDF-15 was reliable in the risk of subclinical coronary atherosclerosis prediction [81]. Perhaps this marker can be promising for the early selection of COPD patients with subclinical coronary atherosclerosis without symptoms of cardiovascular diseases. High serum GDF-15 levels have been reported to be associated with increased risks of mortality and transplantation in patients with pulmonary hypertension (PH) [82]. Accordingly, in another study, GDF-15 serum levels and the soluble suppression of tumorigenicity 2 (sST2) were compared in the AECOPD and AECOPD-PH groups, and the results showed that both serum concentrations were higher in the PH group but there was no relation between hospitalization duration and the levels of GDF15 and sST2 [83].

Soluble urokinase plasminogen activator receptor (suPAR): suPAR is released by the binding of urokinase plasminogen activator (uPA). Active uPA converts plasminogen to plasmin, and it involves in-matrix degradation and activates the complement pathway. suPAR is a potent biomarker of inflammation that is considered a prognostic marker for various inflammatory diseases [84]. Serum suPAR values correlate with several inflammatory markers including CRP, erythrocyte sedimentation rate, fibrinogen, procalcitonin, and white blood cells [85]. Increased suPAR levels are seen in CVD conditions such as ischemic heart disease, stroke, venous thromboembolism, and atrial fibrillation [84]. suPAR can be used as a biomarker for the early detection of COPD, prediction of disease severity, and exacerbation and assessment of treatment response [86,87].

Neopterin: Neopterin is a metabolite of guanosine triphosphate and a biomarker of cellular immunity, systemic inflammation, and CVD. It is released in response to macrophage activation [88]. The Liu Y. et al. study on COPD, asthma, and asthma–COPD overlap patients demonstrated a correlation between increased neopterin levels, vascular dysfunction, and decreased pulmonary function [89]. Moreover, it has been shown that a decline in neopterin level is associated with a reduction in blood pressure and improved vascular endothelial function in hypertensive patients [90]. It appears that elevated levels of neopterin in patients with respiratory disease, due to the risk of decreased respiratory function and vascular disease, require more accurate monitoring.

Sirtuin-1: Sirtuin-1 is a biomarker that plays an important role in nuclease damage and cellular aging [91]. It acts as a nicotinamide adenine dinucleotide (NAD+) -dependent enzyme with different roles in metabolic regulation [87]. Its expression decreases in COPD patients who smoker, and it is associated with accelerated vascular aging and endothelial dysfunction, which lead to CVD [92,93]. The blood level of this biomarker cannot be measured clinically, but due to its role in aging, inflammation, and oxidative stress, which are common COPD and CVD, it can be evaluated as a therapeutic drug target.

Eosinophils: Blood eosinophils play a role in inflammatory processes by releasing inflammatory mediators. They are considered biomarkers for assessing the risk of COPD exacerbation and the response to corticosteroid and biological therapies in these patients [94]. Studies have suggested that higher blood eosinophil counts are associated with higher risk of exacerbation and a better response to inhaled corticosteroid (ICS) treatment. Patients with blood eosinophil levels of 300 cells/µL and above benefit the most from ICS treatment, while if a patient’s blood eosinophil level is less than 100 cells/µL, ICS is not beneficial and should be avoided unless the patient is in severe exacerbation [94]. There is a strong correlation between peripheral blood eosinophils and cardiovascular events in patients with COPD, as eosinophils can accelerate the progression of plaques in coronary arteries by releasing mediators [95].

## 5. Prognostic Value for MACE and All-Cause Mortality

Understanding the mechanisms linking COPD and CVD, as well as identifying the biomarkers that are involved, can provide better insight for effective therapeutic approaches to reducing mortality and morbidity. The Systemic Immune-Inflammation (SII) index, which is based on platelet, neutrophil, and lymphocyte counts, is proposed as a marker of chronic inflammation. In a population-based study, Ye C. et al. indicated that higher SII levels in COPD patients are associated with higher all-cause mortality risk [18]. Platelet activation may be a link between COPD, inflammation, and CVD [31], and so antiplatelet therapy is associated with reduced mortality rate in COPD patients and it alleviates the risk of ischemic events in these patients [96,97].

In the EXACOS-CV US study, Daniels K. et al. showed that COPD exacerbation is associated with an increased risk of acute cardiovascular event and all-cause mortality, and this risk has been noted to be highest in the first 30 days and remains elevated for 1–2 years following each moderate-to-severe exacerbation [98]. Exacerbation prevention is one of the main goals in COPD treatment [40]. Of several studies on inhaled COPD treatments, the IMPACT and ETHOS studies showed that triple therapy including inhaled corticosteroids (ICS)—long-acting beta-agonists—with long-acting muscarinic antagonists as an inhaler was more effective than dual therapy in reducing exacerbation rate, and the addition of ICS as an anti-inflammatory agent was associated with lower mortality rate [99,100]. The UPLIFT trial demonstrated that long-term treatment with inhaled COPD therapy, including tiotropium added to a standard maintenance treatment, was not associated with an increased risk of major adverse cardiovascular event compared with a placebo [101].

Endothelial protective agents such as a phosphodiesterase-4 inhibitor (e.g., roflumilast) have anti-inflammatory effects by preventing the breakdown of cAMP, and they can reduce exacerbation rates in COPD patients [102].

Studies have shown that statins are associated with reduced all-cause mortality in COPD patients in addition to having protective effects against cardiovascular events [103,104]. Fluvastatin and atorvastatin have been associated with lowered CRP levels and pulmonary hypertension in COPD patients [105]. In addition, the RODEO trial showed that the use of rosuvastatin was associated with a reduction in systemic inflammation and an enhancement in endothelial vascular function in COPD patients [106].

## 6. Clinical and Research Implications

Inflammatory biomarkers play a substantial role in linking COPD and cardiovascular disease. They can guide risk stratification, disease monitoring, and personalized treatment.

Many inflammatory biomarkers (e.g., CRP, fibrinogen, etc.) are related to a higher risk of COPD exacerbation and cardiovascular event and can be used to determine the risk of both diseases and assess response to treatment.

Blood eosinophil and fractional exhaled nitric oxide (FeNO) are two biomarkers which are linked to type 2 inflammation, and their role as useful clinical markers has recently been highlighted [107]. The Copenhagen general population study showed that in patients with chronic airway disease, elevated blood eosinophil and FeNO as markers of type 2 inflammation were associated with a faster decline in FEV1 [107]. Eosinophil counts can guide targeted treatments and the evaluation of a patient’s response to treatment. Recent studies have shown that high blood eosinophils are related to moderate-to-severe COPD exacerbations, and high eosinophils have been proposed as a measure of the response to ICS treatment [108].

With a better understanding of biomarkers, targeted biologic therapies have progressed, especially in asthma. In recent studies, these therapies have also been found to be beneficial in COPD. Inflammatory cytokines such as IL13 and IL4 are involved in the recruitment and activation of eosinophils. Trials of dupilumab (a monoclonal antibody against IL13/IL4) have shown an association with reduced annual COPD exacerbation rate and improved lung function in patients with type 2 inflammation [109]. IL8 is another inflammatory cytokine that plays a role in the activation of neutrophils. In a past study, it was proposed as an independent predictor of being a phosphodiesterase-4 (PDE4) inhibitor in COPD patients [110]. Other cytokines like IL5 and IL33 have also been shown to have beneficial effects as targeted therapies for COPD exacerbations [111].

### Strengths and Limitations

This review provides a broad overview of a challenging topic and focuses on reviewing the mechanisms that link COPD and cardiovascular disease, in addition to identifying biomarkers that can be useful in early detection and risk stratification in patients at risk of both diseases. It highlights current evidence and suggests useful guidance for future research. While this narrative review provides a comprehensive literature search, it is not a systematic review, and so it may not be an exhaustive account of all available evidence. This presents the potential for selection bias. Furthermore, heterogeneity in study populations, differences in biomarker measurements in the studies, and a lack of longitudinal data for causal inference are other limitations of this review.

## 7. Conclusions and Future Perspectives

Cardiovascular diseases are important causes of mortality and morbidity in COPD patients. Various mechanisms, including systemic inflammation, oxidative stress, lung hyperinflation, and hypoxia play a role in the connection between these two diseases. Since cardiovascular events are associated with worse prognoses in COPD, recognizing the pathophysiological mechanisms linking these two conditions is crucial, and integrating the management of cardiovascular diseases into COPD care pathways can be effective in reducing the costs to healthcare systems through the early identification of patients at risk, improving the assessment and appropriate treatment of both diseases. Systemic inflammation appears to be an important feature for the pathogenesis of both COPD and CVD, and so identifying specific biomarkers may enable new targeted clinical approaches in screening and treating patients at higher risk of cardiovascular events. Although some novel biomarkers are currently being used in research, potentially, they can be used to identify and monitor high-risk COPD and CVD patients due to their common pathophysiology and also as therapeutic targets in practice.

Considering that COPD and cardiovascular disease are two significant global health burdens and their coexistence can make prognosis and management more complicated, there is a need to move towards more accurate diagnostic and therapeutic strategies. The future of COPD and CVD management is moving towards biomarker-guided strategies and targeted treatments based on specific features, which can be promising for improving outcomes in both diseases.

**Figure 1:**
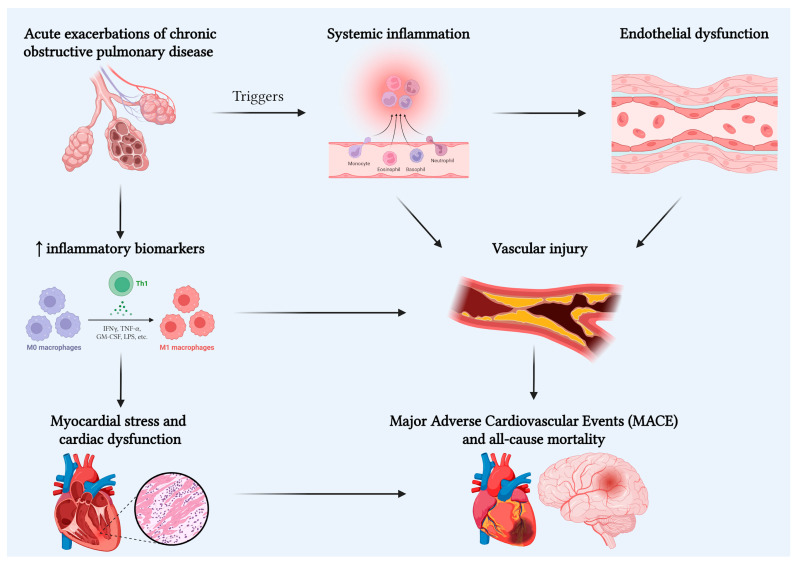
Schematic overview of the hypothesized biological pathway linking the acute exacerbation of chronic obstructive pulmonary disease (AECOPD) to major adverse cardiovascular events (MACE).

Further studies are needed to verify the COPD patients at higher risk of cardiovascular events and novel treatments based on a multidisciplinary approach (Table 1, Figure 1).

## Figures and Tables

**Table 1:** The biomarkers linked to COPD and CVD.

Inflammatory Biomarker	Role in COPD	Role in CVD
CRP	Increased exacerbation risk with decreased lung function	Increased IHD risk
Fibrinogen	Increased risk of mortality and exacerbation risk	Higher IHD risk
BNP/NT-proBNP	Increased disease activity, exacerbation, and mortality risk	Increased levels in heart failure
Troponin	Elevated levels in COPD exacerbation	Detection of myocardial infarction risk
VEGF	Higher levels in COPD exacerbation	Predictor of CVD, promoting thrombogenesis
Surfactant protein D	Increased risk of exacerbation and all-cause mortality	Increased risk of coronary artery disease and atherosclerosis
Galectin-3	Elevated levels in COPD exacerbation	Related to myocardial fibrosis and remodeling
GDF-15	Associated with sarcopenia, higher exacerbation, and mortality rate	Increased levels in CVD
suPAR	Predictor of disease severity, exacerbation, and treatment	Increased levels in CVD
Neopterin	Decreased pulmonary function	Related to the risk of cardiovascular events
Sirtuin-1	Reduced expression in COPD patients who smoker	Related to vascular aging and endothelial dysfunction, possible role in IHD
Blood eosinophil	Predictor of corticosteroid therapy response in COPD exacerbation	Involved in progression of atherosclerosis
ICAM-1	Decreased pulmonary function	Increased risk of atherogenesis

Abbreviations: BNP, brain-type natriuretic peptide; COPD, chronic obstructive pulmonary disease; CRP, C-reactive protein; CVD, cardiovascular diseases; GDF-15, growth differentiation factor-15; ICAM-1, intercellular adhesion molecule 1; IHD, ischemic heart disease; NT-proBNP, N-Terminal proBNP; suPAR, soluble urokinase plasminogen activator receptor; VEGF, vascular endothelial growth factor.

## Data Availability

No new data were created or analyzed in this study. Data sharing is not applicable to this article.
